# Utilizing ChatGPT to Streamline the Generation of Prior Authorization Letters and Enhance Clerical Workflow in Orthopedic Surgery Practice: A Case Report

**DOI:** 10.7759/cureus.49680

**Published:** 2023-11-29

**Authors:** Alioune Diane, Pasquale Gencarelli, James M Lee, Rahul Mittal

**Affiliations:** 1 Department of Orthopaedic Surgery, Rutgers Robert Wood Johnson Medical School, New Brunswick, USA; 2 Department of Orthopaedic Surgery, Orange Orthopaedic Associates, West Orange, USA; 3 Department of Health Informatics, Rutgers School of Health Professions, Newark, USA

**Keywords:** orthopedic surgery, clerical workflow, authorization letter, artificial intelligence in healthcare, chatgpt

## Abstract

Prior authorization is a cumbersome process that requires clinicians to create an individualized letter that includes detailed information about the patient’s medical condition, proposed treatment plan, and any supplemental information required to obtain approval from a patient’s insurance company before any services or procedures may be provided to the patient. However, drafting authorization letters is time-consuming clerical work that not only places an increased administrative burden on orthopedic surgeons and office staff but also concurrently takes time away from patient care. Therefore, there is a need to improve this process by streamlining workflows for healthcare providers in order to prioritize direct patient care. In this report, we present a case utilizing OpenAI's ChatGPT (OpenAI, L.L.C., San Francisco, CA, USA) to draft a prior authorization request letter for the use of matrix-induced autologous chondrocyte implantation to treat a cartilage injury of the knee.

## Introduction

Administrative responsibilities in the physician’s office take significant time and resources that may be better devoted to patient care. The construction of prior authorization letter requests for insurance companies as well as after-visit clinical letters for patients are two prominent examples of such time-consuming and burdensome processes [1]. Prior authorization is a required pre-approval mechanism used by insurance companies to determine what types of treatment are covered by an individual’s insurance policy with the intent to achieve cost-effective and clinically appropriate management. This is thought to encourage the proper use of medical treatments and services, promote patient safety, and manage healthcare spending [1-4]. Similarly, clinical letters are frequently used by orthopedic surgeons to effectively summarize the discussions that took place during the visit, outline immediate next steps in patient care, and provide the patient with details of their overall treatment plan. However, manually formulating these letters is not only time-consuming and resource-intensive but also requires strict attention to detail and adherence to complicated rules imposed by insurance companies [5-9]. Ultimately, this places an increased burden on physician practices and could potentially delay or deny access to necessary medical care, which could in turn result in negative clinical outcomes. This is especially the case with prior authorization letters that fail to adhere to insurance guidelines secondary to administrative errors when manually constructing these letters [4].

The development of large language models such as OpenAI's ChatGPT and GPT-4 (OpenAI, L.L.C., San Francisco, CA, USA) is marketed as "artificial intelligence" (AI) and provides a promising route for easing the burden of clerical labor in healthcare. ChatGPT use in clinical settings is a novel revolution. Thus, there is a need for further literature to demonstrate how healthcare organizations and physician practices may streamline the administrative process and devote more time to patient-centered activities by using the power of ChatGPT's language-generating capabilities. We present a case of prior authorization insurance approval utilizing ChatGPT’s letter-generating capabilities for a 51-year-old female with chronic knee pain who was scheduled to undergo treatment with matrix-induced autologous chondrocyte implantation (MACI).

## Case presentation

The patient is a healthy, active 51-year-old woman with no significant past medical history. She presented to the clinic with a chief complaint of moderate to severe right knee pain. The patient reported that while running a race down a rocky trail, she slipped and twisted her right knee. She reported immediate swelling and pain in her knee. Nonetheless, she was able to finish her race and attempted to manage her symptoms with conservative treatment, including nonsteroidal anti-inflammatory drugs as needed, rest, ice, and bracing.

In the following months, the patient found great difficulty in her ability to perform some of her basic activities of daily living, such as climbing the stairs, kneeling, getting in and out of cars, or even walking her dog down the street. Her right knee pain was relieved with rest. Even after physical therapy and daily activity modification, the patient’s symptoms persisted which prompted her to visit the office for further evaluation. Her past medical history was otherwise unremarkable, and she did not have any history of previous surgeries.

**Figure 1:**
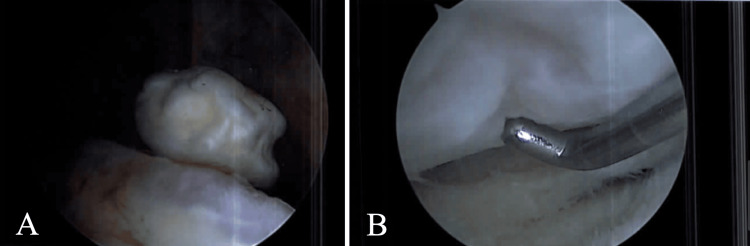
Intraoperative right knee arthroscopic images Intraarticular loose body (1A) and osteochondral defect of the lateral femoral condyle (1B)

A physical exam revealed mild right quadriceps atrophy with weakness, strength 3/5, tenderness to palpation over both the medial joint line and the tibial tubercle, and a slowly healing scar anteriorly. The patient was found to have a full range of motion of the knee and did not exhibit varus/valgus instability. An MRI of her right knee revealed an intraarticularly loose body. After following a short course of physical therapy for quadriceps strengthening, she underwent a diagnostic arthroscopy of the right knee with the removal of a 1.5 cm x 1.5 cm x 0.5 cm loose body and resulting 2x3 cm full-thickness cartilage defect in the weight-bearing surface of the lateral femoral condyle (Figure 1A-1B). Of note, there was no associated arthritis, and the anterior cruciate ligament, posterior cruciate ligament, and medial and lateral meniscus were unremarkable.

**Figure 2:**
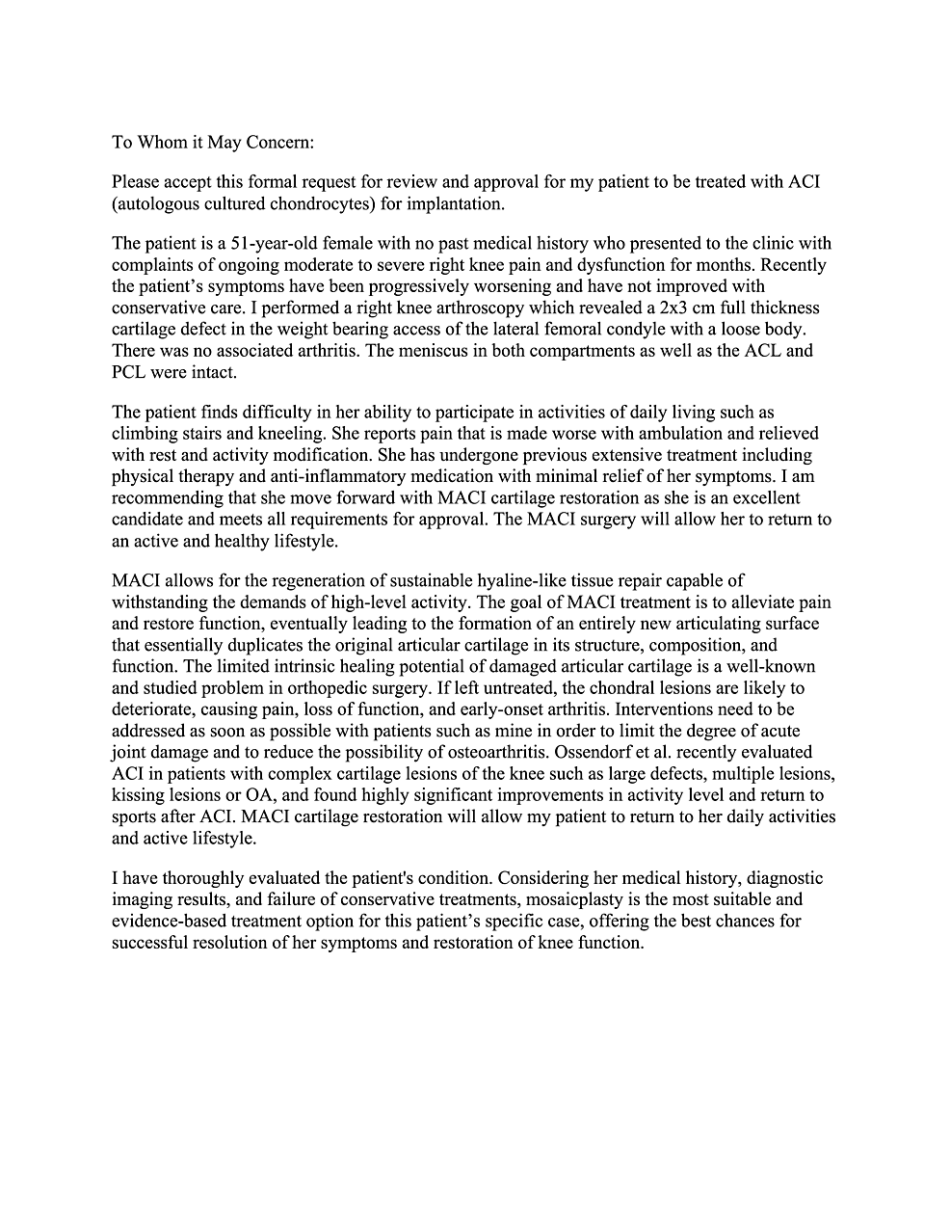
Prior authorization letter to insurance company for MACI procedure ACI: autologous cultured chondrocytes, ACL: anterior cruciate ligament, PCL: posterior cruciate ligament, MACI: matrix-induced autologous chondrocyte implantation, OA: osteoarthritis

To address the osteochondral defect, it was decided to utilize MACI, which offers the patient the ability to utilize autograft restoration (the patient’s own cartilage) as opposed to allograft restoration (donor cells). This procedure is carried out in two stages: the first stage, described previously, is a cartilage biopsy that is sent to the lab for growth/development and checked for viability. The second stage is reimplantation of the autograft cartilage into the osteochondral defect during a second procedure. Of note, the patient was given a full explanation of the treatment rationale prior to proceeding with any further surgical planning. Once the patient agreed with the surgical plan, prior authorization approval from the patient’s insurance company was warranted. The prior authorization letter in Figure 2 was produced by ChatGPT using the following prompt: *“Draft a prior authorization letter to an insurance company coming from an orthopedic surgeon for a patient who will be undergoing matrix-induced autologous chondrocyte implantation (MACI) for the repair of symptomatic cartilage damage of the adult knee, with the goal of alleviating pain, restoring function, eventually leading to the formation of an entirely new articulating surface that will essentially duplicate the original articular cartilage in its structure, composition, and function.”*

The report generated by ChatGPT was reviewed and edited prior to submission to the patient’s insurance company by the senior author. The prior authorization request was ultimately approved, and the team was able to proceed with the two-stage MACI procedure.

## Discussion

When patients present to the office with chronic and severe debilitating pain secondary to a full-thickness cartilage defect in the weight-bearing surface of the lateral femoral condyle, reduction of pain, recovery of function, and return to sport or pain-free activities are the goals of treatment. MACI cartilage restoration has been shown to not only have greater rates of improvement in activities of daily living, quality of life, and pain relief but is also cost-effective when compared to other treatment methods like microfracture [9,10]. On the other hand, delays in cartilage restoration with MACI place patients at risk for deterioration, expanding defects, and the development of new high-grade cartilage defects. Delays prolong patient suffering and potentially impact their postoperative outcomes which may increase overall costs secondary to increased reoperations or increased use of narcotic medications to offset pain [11-13]. While the purpose of prior authorization is to provide high-value treatment, there is debate over how this regulation will affect medical practices and patient access to care. Prior authorization has been shown to significantly increase administrative costs, time, and burden for physician practices in recent countrywide surveys performed by the American Medical Association [14,15]. The surveys also showed prior authorization requests cause delays in receiving critical medical and surgical care, potentially leading to unfavorable clinical outcomes.

Digital platforms for medical professionals are now integrating ChatGPT tools for doctors and offering an entire gamut of workflow solutions to help streamline time-consuming administrative tasks, which contributes to better patient experiences and more time for patient-centered care [16]. Moreover, incorporating ChatGPT into the preparation of prior authorization letters has the potential for significant savings, especially in terms of time and resource efficiency. In a survey administered to 2,802 members of the American Association of Hip and Knee Surgeons, 71% of orthopedic practices employed at least one staff member to solely work on prior authorization who spends on average 15 hours per week [4]. Automating the documentation through the use of ChatGPT would not only considerably speed up the time-consuming process of letter writing but also decrease costs by avoiding the need for hiring additional staff. This time-saving feature is especially important in orthopedic care, where complex surgical procedures and customized treatment programs demand precise attention. The decreased costs can be used to improve other areas of a surgeon’s practice. Furthermore, ChatGPT's ability to seamlessly customize letter templates with patient-specific information, procedural steps, and medical explanations, under the purview of the attending orthopedic surgeon, has boosted the speed with which these letters are generated.

Regarding our patient’s case, using ChatGPT to draft the prior authorization letter for approval by the insurance company did not alter the standard of care delivered to the patient. The use of ChatGPT expedited the overall writing/editing process to less than 10 minutes. Additionally, it provided the office staff with a new option to use and develop going forward. The administrative staff is confident that ChatGPT will help improve administrative efficiency in the practice. At this time, we are unable to determine if using ChatGPT to draft the prior authorization letter increased the probability of approval. However, without its use, the drafting and editing process would have taken the normal one to two hours to complete as opposed to 10 minutes.

Although there are several benefits to utilizing ChatGPT in clinical practice, there are still potential negative drawbacks, including privacy, ethics, bias, discrimination, and validity of information, which must be considered and will need to be addressed in the near future [17]. ChatGPT is subject to falsification/fabrication of content as well as potential bias based on the data on which it is trained [18]. ChatGPT may also lack the ability to comprehend complex medical relationships and is not specifically designed to answer medical questions. ChatGPT is only able to answer questions based on the information it is trained from, and this information may be outdated if not continuously updated.

Since its launch in November 2022, ChatGPT has initially been limited to accessing the up-to-date prior authorization requirements of several health insurance plans. Moving forward, this will be less of an obstacle, as the recent announcement by OpenAI in September 2023 will now allow ChatGPT to move past the September 2021 training data cut-off, which hindered its access to new updates to ICD-10 and procedural terminology codes [19]. ChatGPT has tendencies to generate sources and references that are not valid but may appear plausible, and as a result, it is clinicians’ and healthcare professionals’ responsibility to verify the generated letters/reports from ChatGPT before submitting them to insurance companies for prior authorization.

It is also important to remember that, despite ChatGPT having the potential to revolutionize medical writing, it was not originally designed or tested to provide diagnostic or therapeutic advice. Patient information must still be safeguarded when using ChatGPT, which includes encryption, access control, secure data storage, and compliance with privacy regulations to avoid breaches of privacy or leaks of protected health information [17].

## Conclusions

The use of AI has been garnering much attention, excitement, and steady growth as it continues to play a role in many aspects of everyday life. This is especially the case when it comes to the United States healthcare system, as novel features of AI take on the challenge of solving varied and more complex tasks. Our case highlights how ChatGPT may serve as an effective method for streamlining the clinical letter-writing process in orthopedic clinics, whether that be for prior authorization approval from insurance companies or follow-up instructions for patients. This novel addition may save orthopedic surgeons a significant amount of time, allowing them to focus more on patient care and clinical decision-making. AI-powered technologies like ChatGPT have the potential to transform communication and administrative processes in the healthcare field, warranting further research, development, and potential integration.
